# A High Throughput Single Nucleotide Polymorphism Multiplex Assay for Parentage Assignment in New Zealand Sheep

**DOI:** 10.1371/journal.pone.0093392

**Published:** 2014-04-16

**Authors:** Shannon M. Clarke, Hannah M. Henry, Ken G. Dodds, Timothy W. D. Jowett, Tim R. Manley, Rayna M. Anderson, John C. McEwan

**Affiliations:** Animal Genomics, AgResearch, Invermay Agricultural Centre, Mosgiel, New Zealand; University of Sydney, Australia

## Abstract

Accurate pedigree information is critical to animal breeding systems to ensure the highest rate of genetic gain and management of inbreeding. The abundance of available genomic data, together with development of high throughput genotyping platforms, means that single nucleotide polymorphisms (SNPs) are now the DNA marker of choice for genomic selection studies. Furthermore the superior qualities of SNPs compared to microsatellite markers allows for standardization between laboratories; a property that is crucial for developing an international set of markers for traceability studies. The objective of this study was to develop a high throughput SNP assay for use in the New Zealand sheep industry that gives accurate pedigree assignment and will allow a reduction in breeder input over lambing. This required two phases of development- firstly, a method of extracting quality DNA from ear-punch tissue performed in a high throughput cost efficient manner and secondly a SNP assay that has the ability to assign paternity to progeny resulting from mob mating. A likelihood based approach to infer paternity was used where sires with the highest LOD score (log of the ratio of the likelihood given parentage to likelihood given non-parentage) are assigned. An 84 “parentage SNP panel” was developed that assigned, on average, 99% of progeny to a sire in a problem where there were 3,000 progeny from 120 mob mated sires that included numerous half sib sires. In only 6% of those cases was there another sire with at least a 0.02 probability of paternity. Furthermore dam information (either recorded, or by genotyping possible dams) was absent, highlighting the SNP test’s suitability for paternity testing. Utilization of this parentage SNP assay will allow implementation of progeny testing into large commercial farms where the improved accuracy of sire assignment and genetic evaluations will increase genetic gain in the sheep industry.

## Introduction

To make genetic gain in animal breeding programs, pedigree information is required to estimate breeding values accurately. The use of incorrect pedigree information has the potential to reduce the rate of genetic gain [1], [2]. Furthermore pedigree information is also required for inbreeding management, a crucial element for a successful breeding system resulting in genetic gain [3]–[6].

Traditionally pedigree information has been achieved by breeder records and more recently via DNA marker tests, for example, using microsatellites (MS), also known as either simple sequence repeats or short tandem repeats [7]. However, with the availability of a wealth of genomic information together with development of high throughput genotyping platforms, single nucleotide polymorphisms (SNPs) are now the DNA marker of choice in genomic selection studies. A SNP is a position in the genome that has at least two different bases at that location. These DNA markers are abundant throughout genomes; in sheep there is on average 4.9 SNPs in every 1 kb [8] and 5.1–5.8 SNPs per kb in domestic chickens [9]. These polymorphisms in dog and human are however found at a lower abundance at approximately 1 SNP per kb [10], [11].

The New Zealand (NZ) sheep industry has had the potential to utilize a MS marker test for parentage analysis. Although SNP markers have less polymorphic information (biallelic) compared to MS markers which can have many alleles, this can easily be overcome and superseded by utilizing multiple SNP markers simultaneously. In addition SNPs are superior to MS markers in that, due to utilizing the biallelic SNPs, they are more robust with respect to use in the lab, subsequent interpretation of data and have a lower mutation rate. In addition, short amplicons (<100 bp) can be achieved and high throughput genotyping technologies are applicable to SNP markers. Due to these qualities, we selected a set of SNP markers that were multiplexed with the aim of producing a reproducible, low cost, high throughput genotyping test that is effective in accurately assigning paternity for the NZ sheep industry. Encouraging the use of DNA markers for correct sire assignment will enable accurate breeding values to be estimated and accelerate the rate of genetic gain [1], [2], [12], [13].

To establish a SNP based marker test for sire assignment, substantially more markers are required compared to the traditional MS marker tests. For paternity exclusion, it has been estimated that approximately 4 SNPs with allele frequencies of 0.5 give the same power of exclusion as for 1 MS marker and that variations in the allele frequencies between 0.2–0.8 did not substantially affect the probability of exclusion in paternity cases in human studies [14]. Using the likelihood ratio test for ‘match’ probability, it was found that 50 SNPs with allele frequency of 0.2–0.8 gave the same ratio as 12 MS markers [14]. Several other studies have also determined the number of SNP markers that obtained the same power as MS markers; 59 SNPs in human were found to be equivalent to 13 MS markers [15], 60 SNPs in pigs had similar power to 10 MS markers and in a number of cattle analyses [16] 32 SNPs were found to provide paternity exclusion of 99.9% in trios whereas the same level of confidence was obtained in duos when using 121 SNPs. Fisher et al. [17] found that 40 SNPs alone were more powerful than a typical commercial 14 MS marker panel. A recent study in farmed red deer compared a 12–marker MS panel to one consisting of 100 SNPs where 71% cf. 81% of fawns were matched to their sires, respectively [18]. The authors do, however, note that the difference in performance would have been greater if there had been more SNPs available and that the average SNP minor allele frequency (MAF) had been closer to 0.5. Dodds et al. [19] estimated that three to four times as many SNP markers are needed to achieve the same power as commonly used MS systems, while dominant systems require about 17 times as many markers for assigning both parents. The number of SNPs that are required depends on the MAF and also the scenario the marker panel is to be applied to. Barusch and Weller [20] found that between 15 and 54 SNPs were required to obtain 99% exclusion probabilities. More SNPs were required when the MAF was lower (∼0.1) and less information included (only 1 putative parent genotyped). Parentage identification in sheep via SNP multipex assays have been used in both Australia and North America utilizing 383 and 109 SNPs, respectively [21], [22]. In addition to the use of SNPs for parentage identification and animal verification, the power of SNP markers for traceability have also been investigated [22]–[24].

The practical use of DNA markers to establish a molecular pedigree in livestock breeding programs has a number of issues to address prior to implementation into the industry. If the breeding program is able to sample all parents (i.e. a closed breeding program) and high power of exclusion can be achieved (i.e. many variable loci) without genotyping errors, parents can be identified using Mendelian inheritance of alleles to exclude the incorrect parents. However, the use of pure exclusion methods to assign parentage may fail if information is limiting (where not all parents have been sampled) and genotyping errors are present. To overcome these limitations, several statistical approaches have been developed that rely on likelihood methods whereby probabilities of parentage assignment are determined from simulations or Bayesian methodologies (recently reviewed by [25]). The main differences between these approaches are how the following factors are addressed: genotyping error, calculation of likelihood of the parent-progeny pairs as well as inbreeding and relatedness between parents.

Here, we have developed an Ovine SNP marker panel for parentage assignment and applied it to NZ sheep industry flocks utilizing a likelihood based approach. Furthermore this SNP marker panel can be utilised to infer paternity in the absence of dam information. In addition, a high throughput DNA extraction method from ear-punch tissue has been developed to produce high quality DNA compatible for downstream methods such as Sequenom SNP genotyping, Illumina iScan-bead chip technology and next generation sequencing applications.

## Materials and Methods

### Ethics Statement

The animals used in this study were owned by commercial farmers and managed in accordance with the provisions of the Animal Welfare Act 1999, and the Codes of Welfare developed under sections 68–79 of the Act. DNA sampling was undertaken using tissue collection protocols and standard operating procedures approved by the Invermay Animal Ethics Committee operating under AgResearch's Code of Ethical Conduct for the Use of Animal for Research, Testing and Teaching.

### Animal DNA Samples

The development of the Ovine SNP marker panel utilizing Sequenom iPlex chemistry used DNA samples from a range of NZ breeds including Texel, Romney, Perendale, Booroola and Merino x Romney that had been extracted from heparinised blood using a high-salt method [26]. In total 94 animals, being 34 Sire, Dam and progeny pedigrees (referred to as Trios) were selected for genotyping during the iPlex design process. The animal DNA samples (extracted from both blood and ear-punch tissue samples) for the initial validation of SNP assays following iPlex design were from three internal resource flocks (International Mapping Flock (IMF) (8 sires, 20 dams and 111 progeny); Coopworth flock (12 sires, 37 dams and 39 progeny); Perendale flock (6 sires, 25 dams and 32 progeny). A further validation of the iPlex assay utilized three industry flocks (Flock A, Flock B and Flock C; ear-punch tissue samples) as indicated in Table 1. The breed in both Flock A and C is Romney cross with Flock B containing Poll Dorset cross. In addition the set of dams were available for these flocks, however, the dam of each lamb was unknown. Subsequent beta-testing of the iPlex assay in a commercial lab setting was performed on the DNA extracted from ear-punch tissue from the born 2011 progeny and sires from three industry flocks (Flock D, Flock E and Flock F) from DNA extracted from ear-punch tissue. Flock D contains Poll Dorset cross and both Flock E and F are of composite breed.

**Table 1:** The call rates obtained from genotyping 6 NZ sheep industry flocks with the Parentage SNP assay.

	number of genotypes (produced from 101 SNP assay)	call rate	mismatches
Flock A	47,369	98.67%	0.13%
Flock B	31,411	97.47%	0.29%
Flock C	39,592	98.54%	0.22%
Flock D	357,338	94.98%	0.39%
Flock E	185,739	98.36%	0.27%
Flock F	253,308	97.88%	0.24%
Total/Mean	914,757	97.65%	0.26%

The mismatches (where the assigned sire and progeny do not share any alleles) are also indicated.

### DNA Extraction

Genomic DNA was isolated from blood using a high-salt method as described in [26]. Genomic DNA extracted from ear-punch tissue was isolated by using a modified high throughput version of the Montgomery and Sise [26] method described as follows; tissue samples collected in Tissue Sampling Units (TSU; Allflex, France) were placed into 96 well extraction racks (ThermoScientific AB-1431) and centrifuged at 2500 rpm (Heraeus Multifuge 3 S-R) for 1 min to ensure samples were at the bottom of the tube. An aliquot (200 ul) of proteinase K lysis solution (freshly prepared 20 mg/ml Proteinase K (11.76 µl) added to TNES (188.24 µl; Tris, NaCl, EDTA, SDS: 10 mM Tris pH 7.5, 400 mM NaCl, 100 mM EDTA, 0.6% SDS) was added to the TSUs. The extraction rack was mixed vigorously on an Eppendorf MixMate (1400 rpm; 2 min) prior to overnight digestion at 55°C with gentle shaking (120 rpm). During the first 4–5 hours of incubation, the samples were mixed at 1400 rpm for 2 min (Eppendorf MixMate), 3–4 times to aid in the digestion process. Post digestion, the extraction racks were mixed (Eppendorf MixMate; 1400 rpm, 30 s) and centrifuged at 4000 rpm for 15 min (Heraeus Multifuge 3 S-R). Supernatant (150 µl) was transferred to a 0.8 ml V bottom 96 well storage plates (ThermoScientific AB-0765) and 42 µl 5M NaCl was added, mixed by firmly shaking for 1 min prior to centrifugation at 4000 rpm for 30 min (Heraeus Multifuge 3 S-R). Supernatant (150 µl) was transferred to another 0.8 ml V bottom 96 well storage plates (ThermoScientific AB-0765) with an equal volume Analar Ethanol (Merck, Darmstadt, Germany) added. The plates were sealed, inverted several times and allowed to sit at −20°C for at least one hour. Following precipitation, the plate was centrifuged at 4000 rpm for 30 min (Heraeus Multifuge 3 S-R) and ethanol decanted without disturbing the DNA pellet. The DNA pellet was washed with 70% ethanol (Merck, Darmstadt, Germany; 500 µl added to the plate, sealed and inverted several times and incubated at room temperature for at least an hour). The plates were centrifuged at 4000 rpm for 15 min (Heraeus Multifuge 3 S-R) and ethanol decanted without disturbing the pellet. The DNA was air dried prior to eluting in 200 µl TE (10 mM Tris-HCI (pH 8.0), 1 mM EDTA (pH 8.0)). All chemicals were from Merck KGaA, (Darmstadt, Germany) unless otherwise stated. The DNA samples were quantified using the Nanodrop8000 (ThermoFisher Scientific Inc).

### SNP Sequences and Assay Design

The SNP sequences that were considered for inclusion in the Parentage SNP assay had been previously validated on both the Ovine 1536 Golden Gate SNP assay and the Ovine SNP 50K Illumina chip (n = 454). To establish the SNP set to be submitted to Sequenom Inc. (Australia), the SNPs were given an index value (*I_j_*  =  Σ_b_(MAF_bj_–0.5)^2^ for the *j*th SNP, where MAF_bj_ is the allele frequency in the *b*th breed) in the HapMap samples genotyped with the 1536 SNP assay [8]. The 300 SNPs with the smallest *I_j_* value together with SNPs that had a MAF>0.25 for Romney, Perendale and Coopworth animals (n = 250) were subjected to preliminary assay design (MassARRAY software) to select the top 300 SNPs that would be submitted to the Sequenom iPlex assay development process. The location of the SNPs was also considered so that relatively equal chromosome spacing throughout the genome would be achieved. In addition to the 300 proposed Parentage SNPs, 19 “production trait” SNPs were also submitted to Sequenom (San Diego, CA) for inclusion in the iPlex assay along with the Trio animals supplied as a 50 µl sample (20 ng/µl) in a 96 well plate for development of a parentage SNP assay. The various steps of the design process were validated by genotyping the Trio animal set. The resulting parentage SNP plus production SNP Sequenom iPlex assay, hereafter referred to as the Parentage SNP assay, that was generated by Sequenom consists of a two plex 115 SNP (58 and 57 SNPs) product. Information on the 98 parentage SNPs in the Parentage SNP assay are located in Table S1 with the iPlex assay design located in Table S2. This Parentage SNP assay was then subsequently validated by AgResearch utilizing internal research flocks followed by beta testing in industry flocks to assess the suitability of the 98 parentage SNPs and the 17 production SNPs. Following the genotyping of the research flocks, Mendelian inheritance errors, indicating allelic dropout, were detected in 12 SNPs and these SNPs have been excluded from the parentage analysis. These same SNPs also typically had lower call rates and extreme Hardy Weinberg values. A further 2 SNPs were also found to give Mendelian inheritance errors after beta testing in the industry flocks. These SNPs have also been removed from subsequent genotype and parentage analysis. In total 84 parentage SNPs are suitable for use in the Parentage SNP assay (Table S1). The 17 production SNPs are not discussed further in this study. Reproducibility of the SNP multiplex assay was assessed initially by comparing genotype results obtained by AgResearch with those from Sequenom for the Trio animal set utilized for development. Further reproducibility was investigated in the commercial genotyping laboratory environment where the same three samples were genotyped in 6 independent assay runs.

To provide further information to the international community, SNPs were identified in the 100 base pair flanking regions of the 98 parentage SNPs for the International Sheep Genomics Consortium (ISGC) diversity panel of 73 sheep samples that were sequenced to ∼12 X coverage (www.sheephapmap.org). These are presented in Table S3.

### Sequenom iPlex Chemistry Assay

All DNA samples (12.5 ng/µl) were transferred into 384 well PCR plates for genotyping. They were arranged by plex and are referred to as Plex 1 (58 SNPs) and Plex 2 (57 SNPs). The oligonucleotides were supplied by Sequenom as combined primer pools (Plex 1 and Plex 2, Amplification and iPLEX). The oligonucleotide sequences for the 98 parentage SNPs are located in Table S2. The genotyping analysis was performed as recommended by the manufacturer with reagents included in the iPLEX Gold SNP genotyping kit (Sequenom) and the software and equipment provided with the MassARRAY platform (Sequenom). Plex 1 and Plex 2 were amplified from a 5 µl final PCR volume composed of 1×PCR buffer, 2 mM MgCl_2_, 500 µM deoxynucleotide triphosphates (dNTPs), 0.1 µM each PCR primer, 0.5 U of HotStarTaq enzyme, and 1.5 µl DNA. The thermal cycling conditions consisted of a first denaturation step at 95°C for 2 min, followed by 45 cycles of denaturation at 95°C for 30 s, annealing at 56°C for 30 s, and extension at 72°C for 1 min, with a final extension step at 72°C for 5 min. To neutralize unincorporated dNTPs, PCR products were treated with 0.5 U shrimp alkaline phosphatase by incubation at 37°C for 40 min, followed by enzyme inactivation by heating at 85°C for 5 min. By adding 2 µl of an iPLEX Gold extension reaction cocktail to the purified PCR products, the Plex 1 and Plex 2 extension reaction was carried out in a final volume of 9 µl containing 0.222×iPLEX buffer, 1×iPLEX termination mix, 1×iPLEX enzyme, and the SBE primer mix that contained the Plex 1 and Plex 2 extension primers. The iPLEX extension reaction was performed under the following thermal conditions: an initial denaturation step at 94°C for 30 s, followed by 40 cycles of a denaturation step at 94°C for 5 s, 5 cycles of annealing at 52°C for 5 s and extension at 80°C for 5 s and a final extension step at 72°C for 3 min. After desalting of the products by using SpectroCLEAN resins following the manufacturer’s protocol, cleaned extension products were dispensed onto a 96 SpectroCHIP array using an RS1000 Nanodispenser, and finally, the array was introduced into a MassARRAY Compact 96 mass spectrometer. Spectra were acquired using SpectroAcquire software, and data analysis, including automated allele calling, was done using MassARRAY Typer software, version 4.0.5.

### Paternity Assignment

The first approach taken to assign paternity in this study is based on the methods described by Marshal et al. [27] and Dodds et al. [28] utilizing likelihood ratios and parentage probability (referred to hereafter as AgR method). The allele frequencies were estimated using a weighted average of those in the progeny (0.05 weighting) and in the potential parents (0.95 weighting), but with a minimum allele frequency of 0.01. The error rate was set to 0.5%. To infer paternity, LOD scores (log of the ratio of the likelihood given parentage to likelihood given non-parentage) were calculated for each possible parentage. Only those with a LOD score of at least zero, and paternity probability [28] of at least 0.02 were retained for further consideration. Parentage was assigned to the sire with the highest LOD score. A sire is not assigned if the best LOD ≤0. A Δ statistic was also utilized to resolve paternity [27]. The Δ values were determined as a measure of how close other possible parentages were to the best parentage and are calculated by subtracting the LOD of the 2^nd^ best sire match (LOD2) from that of the best Sire match (LOD1). Note that for this first approach if the 2^nd^ best LOD is <0 then Δ is defined to be LOD1 (rather than LOD1-LOD2). This differs from that of the Cervus 3.03 program also utilized in this study where LOD2 is used even if <0.

For the paternity assignment using likelihood methods implemented through the Cervus 3.03 computer program, allele frequencies were determined as above for each locus prior to performing simulation analyses to establish a confidence threshold for assignment. This software package is marketed as an easy-to-use and practical tool to establish parent-offspring relationships when some genotypes are incomplete, incorrect or missing. It assumes that markers are autosomal and that the species is diploid. It also assumes that markers are inherited independently of each other (not in linkage disequilibrium). In the simulation, the identity of the true parent is known for each offspring. Cervus compares the distribution of LOD or Δ scores for tests in which the most likely candidate parent is the true parent with the distribution of LOD or Δ scores for tests in which the most likely candidate parent is not the true parent. Confidence in assignment is defined as the proportion of all candidate parents with LOD or Δ scores exceeding a given LOD or Δ score and are the best LOD or Δ that are true parents [27]. The 99% and 95% critical LOD and Δ values shown on the Δ-LOD plots in this study were derived from simulations involving 10,000 offspring. A 0.5% genotyping error was assumed (as in the method above) and likelihood was calculated using the equations of Marshal et al. [27]. For the purposes of the analysis, it was assumed that the proportion of candidate parents sampled was 0.95. Sensitivity analysis showed that the selection of this parameter in the range of 0.9 to 1.0 had very little effect on the proportion of offspring with parentage assigned at different levels of confidence.

A simulation was also carried out using the genotypes from Flock D assuming each sire produced the number of progeny that was estimated from the initial analysis. The progeny genotypes were generated by randomly sampling alleles from the true genotypes of the sires and the alleles from the dams were obtained by random sampling based on the proportions given in the allele frequency files. The simulated genotypes for the offspring were then combined with the true genotypes of the sires and modified to mimic the missing and mistyped loci observed in the original data. These genotypes were then processed through Cervus using the same approach as for the real genotype data.

## Results

### High Throughput DNA Extraction Method

The high throughput ear-punch tissue DNA extraction method established produces good quality and quantity of DNA suitable for various downstream genotyping platforms including next generation sequencing technologies. On average, 36 µg of DNA (mean 260/280 ratio = 1.81) was extracted from an ear-punch tissue collected in an AllFlex TSU. When genotyped using the Sequenom system call rates of 96–98% were obtained. In addition to utilizing the DNA extraction method for the Sequenom Parentage SNP iPlex assay developed in this study, the extracted DNA has also been used to genotype samples with the Illumina Ovine 5K SNP chip (n = 2,232). When the accepted Illumina genotype call rate was set to greater than 95%, 0.4% of samples failed and when a more stringent call rate of at least 98% was set, 1.5% failed (data not shown). Furthermore, the DNA is suitable for both whole genome and reduced representational next generation sequencing techniques. Sequencing Tru-seq paired end libraries on the HiSeq 2000 produced an average of 48 Gb of raw sequence/lane which when trimmed (DynamicTrim with default settings; removes all bases with a quality score less than 13) reduced to 41 Gb of sequence (Figure S1). For the reduced representational sequencing, 1,672 ear-punch tissue sample extracted DNA have been utilized in a genotyping-by-sequencing method producing average data sets of 35 Gb/lane (data not shown).

### SNP Selection and Assay Development

The developments in iPLEX Sequenom chemistry for multiplexing of up to 60 SNPs in a single multiplex allow for a cost competitive high throughput parentage assay to be established. Following the selection of 300 SNPs it was anticipated that 2 iPLEX assays of approximately 60 SNPs each would be sufficient for paternity assignment. The resulting SNPs in the Parentage SNP assay are autosomal and span all but chromosome 16 and 26 (Table S1). There were no X or Y SNPs present, therefore sex cannot be determined when using this assay.

The initial validation of the Parentage SNP assay identified 12 SNPs that gave pedigree errors. The DNA samples used for the initial validation had been extracted from both whole blood and ear-punch tissue with the resulting call rates of 99% and 96–98% obtained, respectively. The reproducibility of this test was 99.8%. The concordance level between the Sequenom iPLEX and the Illumina platforms (n = 126 animals) was found to be 96.1% for the 98 parentage SNPs in common between both platforms. However, upon removal of the 14 non-performing SNPs (12 identified during initial validation with an additional 2 SNPs identified during the beta test; Table S1) the concordance between the genotyping platforms increased to 99.2%.

### Assay Performance

The call rates obtained from genotyping 6 industry flocks with the Parentage SNP assay (84 parentage +17 production SNPs) are presented in Table 1. The average call rate obtained was 97.7% with 0.26% mismatches (where the assigned sire and progeny do not share any alleles). The lowest call rate obtained was 95% for Flock D, however, this was subsequently determined to be caused by a technical issue during the DNA extraction procedure prior to genotyping.

**Figure 1:**
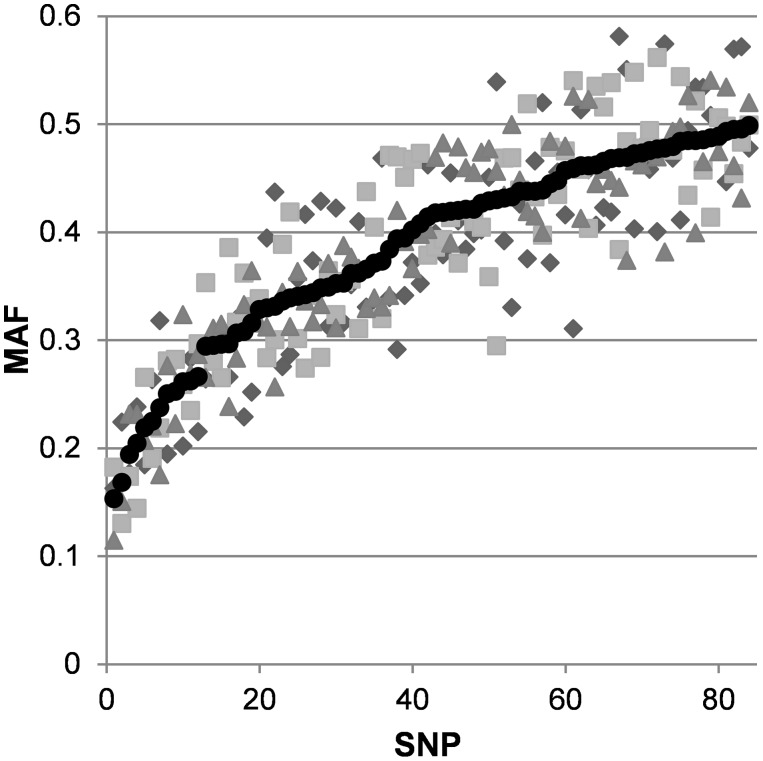
The minor allele frequency (MAF) of the 84 Parentage SNPs observed in three industry flocks. The average MAF is indicated by the black circles with Flock D (squares), Flock E (diamonds) and Flock F (triangles). The minor allele was determined from the average MAF.

The Parentage SNPs selected for assay design were required to have an estimated MAF greater than 0.25 in the predominant NZ breeds; Romney, Perendale and Coopworth. The observed MAF following analysis of the parentage SNP genotypes obtained from Flocks D, E and F during the beta test are presented in Figure 1. Only 10 of the 84 Parentage SNPs were found to have an average MAF<0.25. Over 50% of the parentage SNPs had a MAF greater than 0.4. In addition, the polymorphic information content (PIC; [29]) was also calculated where the range of PIC was found to be 0.23–0.38 (Table S1).

### Paternity Assignment: Initial Validation

The initial validation of the Parentage SNP assay found 12 SNPs to be suboptimal and they have been removed from further analysis. In addition, a further 2 SNPs were also removed from subsequent analyses following the beta test in a commercial lab environment (these had >2% mismatches for assigned pedigrees in 5 of the 6 flocks). Summary statistics for the sire assignment-best pedigrees (i.e. the sire with the highest parentage probability) for the industry flocks utilized in the initial validation are presented in Table 2 with or without dam information included. When both the dam and sire genotypes were included in the analyses, the average best parentage probability was found to be 0.99 with a LOD of 32.41 (Table 2). On average, 98.8% of the progeny were assigned where 97% had unique assignment. Furthermore an average Δ, which represents the difference between the best and the 2^nd^ best parentage assignments, of 31.91 was obtained (Table 2). When paternity was assigned to the progeny using only the sire information, the LOD scores reduced substantially as did the Δ values with an average of 11 and 10.9 obtained, respectively (Table 2). Although the percentage of progeny that could be assigned did not change in the sire only analysis, the percentage that were uniquely assigned decreased by 9% for Flock A and 12% for Flock B, with a corresponding decrease of 0.09 in the assigned sire probability for these two flocks. For Flock C, the assigned sire probability and proportion of unique assignments in the sire only analysis remained similar to that for the analysis including dams.

**Table 2:** Summary (% or mean ± sd) of paternity assignment (AgR method; Best Pedigree^a^) results in 3 industry flocks.

Number Genotyped^b^				Sire Assigned (Dam genotypes included)					Sire Assigned (Sire genotypes Only)				
Flock	Sires	Dams	Progeny	%	LOD	Prob	Unique	Δ	%	LOD	Prob	Unique	Δ
A	22	174	314	100	31.44±8.79	0.99±0.06	0.98±0.14	31.03±9.56	99.6	9.79±4.54	0.90±0.28	0.89±0.32	9.75±4.59
B	13	125	173	100	33.41±9.74	0.99±0.05	0.97±0.18	32.84±10.94	98.3	10.99±6.47	0.90±0.26	0.85±0.36	10.83±6.63
C	27	135	240	96.3	32.39±10.5	0.99±0.06	0.97±0.18	32.15±11.06	98.3	12.22±5.46	0.99±0.06	0.96±0.20	12.12±5.57
Average				98.8	32.41	0.99	0.97	31.91	98.7	11.00	0.93	0.90	10.9

a Best pedigree refers to the pedigree with the highest assignment probability (Prob) for a particular analysis. In addition the LOD (LOD is the log of ratio of likelihoods under correct and incorrect parentage), uniqueness (proportion with a unique assignment) and Δ (the LOD difference between the best and next best assignment) is also recorded. ^b^A subset of each industry flock were genotyped along with the potential sires and dams for the whole flock.

In addition to genotyping Flock A with the Parentage SNP assay, the genotypes from a commercial MS marker test were also available for these animals and analyses compared and presented in Table 3. This MS test consists of 11 markers (PIC values ranged from 0.65 to 0.78) and was designed for assigning both the dam and sire not just for paternity assignment. To establish a ‘Gold Standard’ for the comparison of assignment results, as the true parentage is unknown, a combined analysis was performed using both MS and SNP markers as well as including the dam, the lambing and mating mob information. Analysis containing only the MS resulted in a best parentage probability of 0.88 and a mean LOD of 15.44. A 0.11 increase in the parentage probability and a doubling of mean LOD were observed when utilizing the SNP markers alone compared to MS only. The ‘Gold Standard’ analysis resulted in a further increase in the mean LOD (Table 3). However, the increased power of the Parentage SNP assay compared to MS markers alone, is most evident from both the ‘uniqueness’ and Δ values reported in Table 3. Only half were reported to be unique for the MS test, however, this increased to 98% for the Parentage SNPs analysis. For the Δ value, a mean difference of 9.25 was observed for the MS analysis compared to 31.03 for the Parentage SNPs only (Table 3). The Δ value further increased with the ‘Gold Standard’ analysis to 43.10. There were 82.0% and 97.7% correct parentage assignments for the MS analysis and selected SNPs, respectively, assuming the Gold Standard results were 100% correct (data not shown). The same analyses were also carried out with sire only matching and results presented in Table 3. Only 37% unique assignment was reported for the MS test alone. This improved with a unique assignment of 89% for the SNPs analysis. As with the parentage analysis presented above, this paternity analysis also highlighted the increased power of the SNP based genotyping compared to the MS test.

**Table 3:** Summary (% or mean ± sd) of paternity assignment comparison (AgR method; Best Pedigree^a^) of Flock A utilizing MS markers, Parentage SNPs and of a combined marker analysis (Gold Standard).

Test^b^	Assignment-Dam and Sire included					Assignment-Sire Only				
	%	LOD	Prob	Unique	Δ	%	LOD	Prob	Unique	Δ
MS	100	15.44±2.71	0.88±0.18	0.50±0.50	9.25±7.57	99.7	4.82±1.71	0.82±0.19	0.37±0.48	3.18±2.46
SNP	100	31.44±8.79	0.99±0.06	0.98±0.14	31.03±9.56	99.6	9.79±4.54	0.98±0.06	0.89±0.32	9.75±4.59
combined	99.7	43.70±13.81	0.99±0.07	0.95±0.22	43.10±15.25					

a Best pedigree refers to the pedigree with the highest assignment probability (Prob) for a particular analysis. In addition the LOD (LOD is the log of ratio (likelihood correct/incorrect), uniqueness (proportion with a unique assignment) and Δ (the LOD difference between the best and next best assignment) is also recorded. ^b^The MS test consists of 11 SSR markers, the SNP test consists of the 84 Parentage SNP markers and the combined test has both marker sets and is referred to as the ‘Gold Standard’. The mob data is also utilized in the combined ‘Gold Standard’ analysis along with the Dam genotypes.

In addition, as a measure of the relative power of the marker sets, exclusion probabilities [7] were calculated using the allele frequencies from this flock. The probabilities of excluding an incorrect sire, with dam unknown, were 0. 9944 for the MS set and 0.9998 for the SNP set (Table 4). The expected frequencies of incorrect matches using the SNP set are one in 5600 when paternity testing in the absence of dam information, one in 5.5 million when paternity testing with known dam genotype information, and 1 in 79 billion when testing putative parent pairs. Approximately 50 SNPs (with average exclusion power for the SNP set) were required to achieve the same exclusion power as the set of 11 MS markers.

**Table 4:** Exclusion probabilities calculated for Flock A for the set of 11 MS markers and 84 Parentage SNPs.

Test	Progeny - Parent Pair (Q3)^a^	Progeny - Single Parent, other parent genotype known (Q1)^a^	Progeny - Single Parent, other parent genotype unknown (Q2)^a^
MS	0.999999719	0.999864311	0.994429832
SNP	1.000000000	0.999999817	0.999822454

a Q1, Q2 and Q3 are the notation used for these exclusion probabilities in [7].

### Paternity Assignment in Three Industry Flocks

A total of 7,875 animals were genotyped (252 sires and 7,623 progeny) from three industry flocks where the genotype call rate ranged between 94.98% and 98.36% (Table 1) with assignment of 98.8% progeny to sires (Table 5) and mean sire probability of 0.98 were obtained when processed through a commercial lab environment.

**Table 5:** Summary (% or mean ± sd) of paternity assignment (AgR method; Best Pedigree^a^) of 3 industry flocks.

Number Genotyped			Sire Assignment-best match				
Flock	Sires	Progeny	%	LOD	Prob	Unique	Δ
D	125	3403	99.0	11.90±4.50	0.97±0.10	0.87±0.33	11.34±5.64
E	52	1787	97.8	12.97±5.27	0.99±0.05	0.95±0.21	12.87±5.43
F	75	2433	99.5	14.96±5.87	0.99±0.05	0.96±0.20	14.81±6.08
		Average	98.8	13.28	0.98	0.93	13.01

a Best pedigree refers to the pedigree with the highest assignment probability (Prob) for a particular analysis. In addition the LOD (LOD is the log of ratio (likelihood correct/incorrect), uniqueness (proportion with a unique assignment) and Δ (the LOD difference between the best and next best assignment) is also recorded.

To establish a confidence threshold for assignment, parentage inference was performed on the six data sets from Flocks A–F using the likelihood methods implemented in Cervus. Similar to the AgR method, Cervus also calculates likelihood ratios allowing for the possibility that the genotypes of parents and offspring may be mistyped.

**Figure 2:**
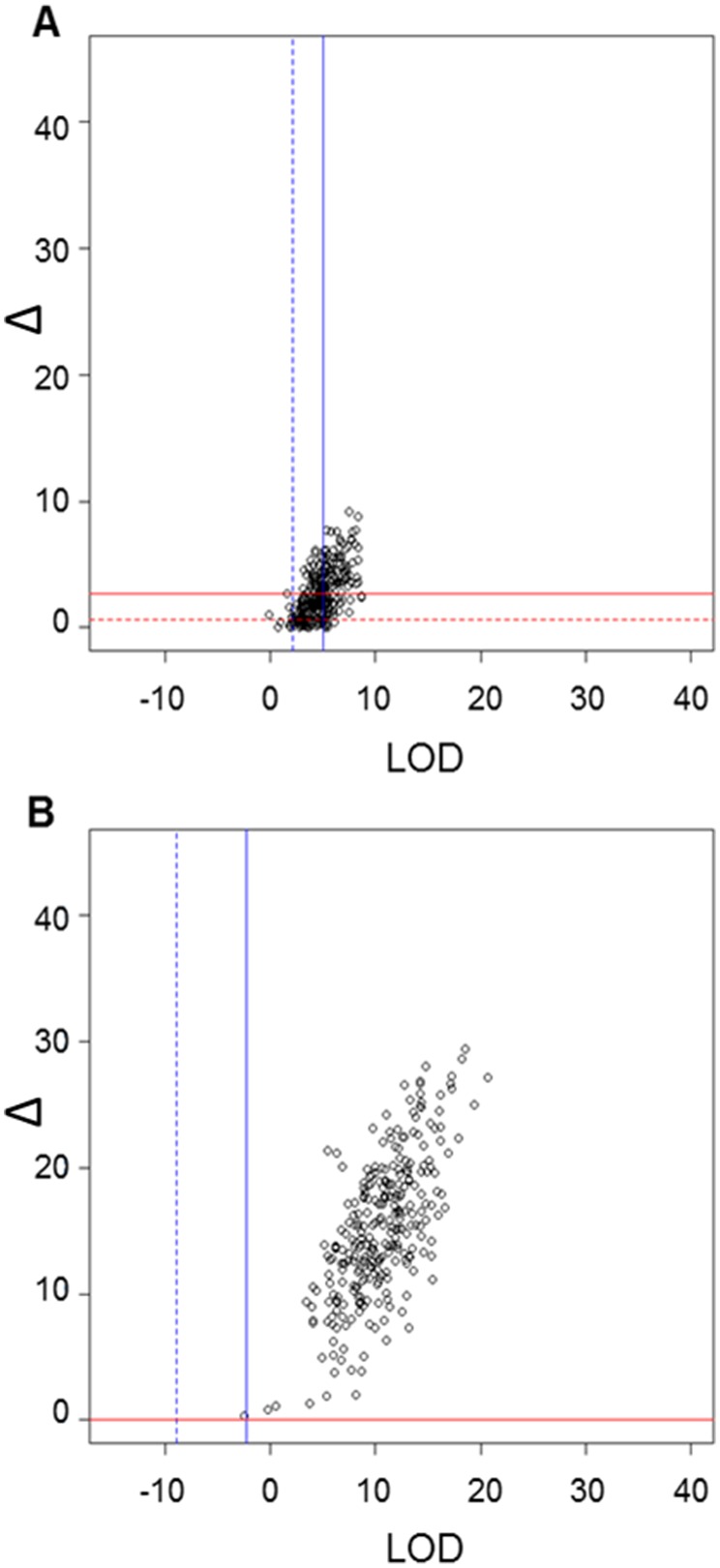
Comparison of paternity assignment utilizing genotypes determined from 11 MS markers or 84 Parentage SNP assay. The sires and progeny from Flock A were genotyped with either MS markers (A) or Parentage SNP assay (B) and paternity was assigned using Cervus. A simulation analysis was performed to allow for the evaluation of the confidence in assignment of parentage to the most likely candidate parent where the 99% (solid line) and 95% (dashed line) critical LOD and Δ values shown as blue or red lines, respectively. The black circles represent the Δ and LOD values obtained for each of progeny.

**Figure 3:**
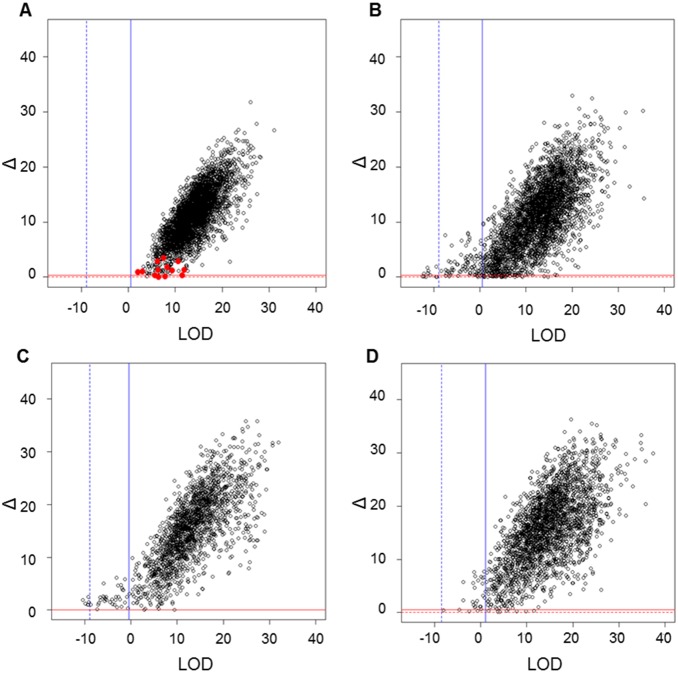
Paternity assignment of progeny from Flocks D–F genotyped with Parentage SNP assay as determined by Cervus. A simulation analysis was performed to allow for the evaluation of the confidence in assignment of parentage to the most likely candidate parent where the 99% (solid line) and 95% (dashed line) critical LOD and Δ values shown as blue or red lines, respectively. The black circles represent the Δ and LOD values obtained from true genotypes for each of progeny for Flock D (B), Flock E (C) and Flock F (D). In (A) the results of a simulated parentage assignment using data from Flock D are presented. The red dots in the first plot mark the offspring with incorrectly allocated sires.

The results for Flock A from the Cervus analysis are presented in Figure 2 indicating the critical cut-off values for both the 95% and 99% confidence in assignment of parentage to the most likely candidate parent, as determined by simulation. This figure compares the MS markers to that of the Parentage SNP assay. At the 99% level, more than half (55%) of the sire assignments fell below the critical Δ or LOD values set to confidently assign paternity when genotyped with the MS markers. However, only 0.35% of assignments were not confidently assigned using the Parentage SNP genotypes. The results of analysing simulated genotypes from Flock D with Cervus, using the same approach as for the true genotype data, are presented in Figure 3A. By comparing the known sires with most likely sires, it was found that only 13 out of 3,397 offspring were assigned incorrect sires at the 99% confidence level. This equates to an error rate of 0.38%. The sire assignment for Flock D using the true genotypes is presented in Figure 3B where, at the 99% confidence level, 6% of progeny were not confidently assigned a sire. Only 2% of the progeny from both Flock E and F using the true genotypes fell below the critical Δ and LOD values set to infer paternity (Figure 3C–D). The 4% higher unassigned progeny for Flock D maybe a direct result of the lower genotyping call rate obtained for these progeny (95% cf. the 98% average call rate for the other 5 flocks). When comparing the assignment from the AgR and Cervus methods, between 96–100% for progeny from Flocks A to F gave the same results for the Parentage SNP assay compared to 93% of the progeny for Flock A obtaining the same results for the MS markers test (data not shown).

**Figure 4:**
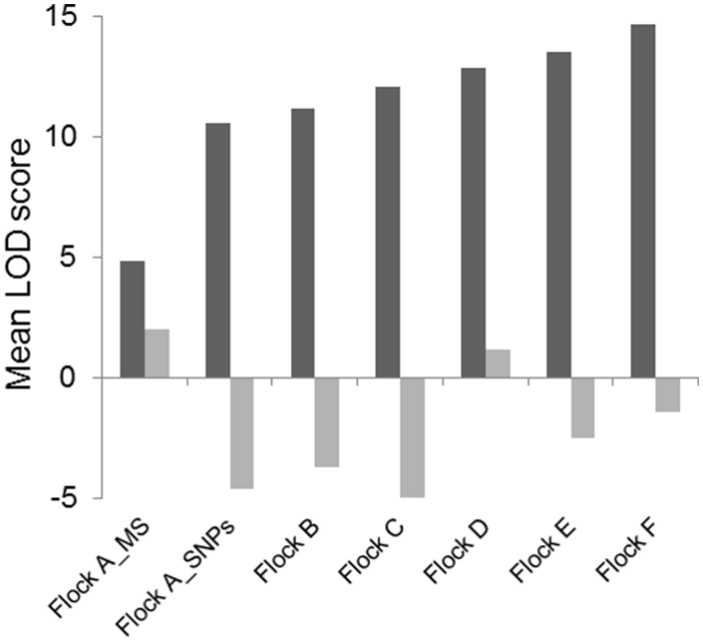
The mean LOD score (the log of ratio (likelihood correct/incorrect)) of the best (black bars) and 2^nd^ best (grey bars). Sire assignment for the six industry Flocks A–F was determined by Cervus. Flock A has been genotyped with both the MS and the 84 SNP Parentage assay.

To further illustrate the superior power of the Parentage SNP assay compared to the MS markers in paternity assignment, the mean LOD scores for the best and 2^nd^ best sire assignments are presented in Figure 4. The mean LOD scores for the best sire assignments are all positive for each flock; however, they are also positive for the 2^nd^ best sire assignment when genotyped with the MS markers (Flock A) and also for Flock D using the SNP markers. The latter result may be indicative of the lower genotype call rate obtained for this flock or that there is higher degree of relatedness between putative sires in this flock compared to the others. The mean LOD score for the 2^nd^ most likely sire in the other flocks and also Flock A using SNP markers is negative.

## Discussion

The aim of this study was to produce a high throughput, reproducible, cost effective and accurate parentage test for implementation into the NZ Sheep Industry. The use of DNA markers to establish parentage, a requirement for estimating breeding values accurately and managing inbreeding, has aided in the progress of genetic gain in animal breeding programs. Misidentification rates of 7–15% have been reported which have led to a 2.5% to 15% decrease in genetic gain [1], [30]–[34]. The sheep industry in NZ has relied on MS markers which when used together with implemented farm management systems such as mob recording [35], has provided adequate parentage for breeders. However the ability of the current MS markers to assign parentage correctly with high confidence is substantially less than that observed for SNP marker based tests, assuming sufficient markers are included in the assay.

In this study, the power of the Parentage SNP assay was found to be superior to that of the MS marker test alone. This is to be expected as the Parentage SNP assay has 34 more SNPs than the number estimated to achieve the same power as the MS marker test (∼50 SNPs were required to achieve the same exclusion power as set of 11 MS markers). This estimation of 4.5x SNPs to one MS marker is in agreement with previous studies [14]–[19]. Nevertheless, the aim of the study was to produce a high throughput, reproducible and cost competitive SNP based assay that has the power to assign paternity without additional on-farm information. The alternative would be to introduce additional MS markers, via an additional plex to the current test to achieve the same power of assignment observed for the Parentage SNP assay. Although this may result in fractionally lower assay cost, this would be the only advantage because the SNP marker panel is also a subset of other larger SNP panels (eg Illumina Ovine SNP arrays 5K, 50K and HD). This attribute avoids the need for re-genotyping any potential sires that are already genotyped using one of these panels. In addition, the ability of SNP markers to be easily standardised across laboratories allows for generation of international SNP panels for traceability as discussed below.

The observed increased power of the Parentage SNP assay is most evident from the ‘uniqueness’ values where, when both the dam and sire information was also included in the analysis of the flock examined, only 50% of progeny assigned were reported to be unique for the MS test, however, this increased to 99% for the SNPs analysis. For paternity only assignment situations, the MS test often reported multiple possible paternities (only 37% unique assignment) which greatly improved to a unique assignment of 89% for the SNPs assay, further highlighting the higher power of the latter test. A similar finding was also observed when the Cervus program was utilized to assign paternity at a given confidence level. More than half of the progeny could not be assigned at the 99% confidence level when genotyped with the MS markers compared to less than 1% for the SNP markers.

The use of DNA markers in extensively farmed livestock have generally been used to either verify or exclude potential parentage. In this study categorical assignment has been utilized that can be used when parentage information is incomplete, allows for genotyping errors, and is able to assign putative paternity (and also parentage pairs) [28]. The Parentage SNP assay developed here was found to be reproducible and routinely produce call rates ≥96% in a high throughput commercial genotyping lab highlighting the robustness of the test. Cooper et al. [36] investigated the relationship of call rate and accuracy in SNP genotyping (BovineSNP50 and BovineLD) in dairy cattle and recommend edits on call rates so as to reduce the use of incorrect genotypes. They found that call rates ≤80% resulted in unreliable parentage verification in duo tests whereas with call rates ≤90% validation of parentage in trios were not reliable.

The initial validation carried out on Flocks A to C provided evidence for the power of unique assignment to sire in a flock of up to 300 lambs from mob mating of rams. The finding suggested that flock management and/or recording systems could be altered to reduce breeder involvement. To further investigate this, several large paternity test situations were undertaken, without knowledge of dam genotype or lambing or mating groups. The progeny and potential sires from three industry Flocks (D, E and F) were genotyped with the Parentage SNP assay in a commercial lab environment with subsequent paternity assigned. On average 99% of progeny were assigned a sire with 93% uniquely assigned with an average Δ value of 13.01 obtained using the AgR method. These results indicate that the SNP based parentage test has the power to uniquely assign 3,000 progeny that result from mob mating and without additional information such as the subset of all sires that the true sire belonged to (which might be known if different mating groups were retained to lambing). Dam information (either recorded, or by genotyping possible dams) was unavailable during this validation phase, highlighting the SNP test’s suitability for paternity testing. When the same data sets were analysed using the Cervus software program between 93.8 and 98.4% of the progeny were confidently assigned a Sire at the 99% confidence level.

The use of categorical assignment of paternity in this study using the highest likelihood of putative parent-progeny pairs given their multiple SNP genotypes and observed allele frequencies in the flock has resulted in at least 98% of progeny assigned to a sire. The accuracy of parentage assignment analysis tests differ depending on factors such as the number of loci and polymorphic content, ability to sample all candidate parents and the presence of genotyping errors. Recently the relative accuracy of different methods of parentage analysis was investigated, albeit in natural populations using MS markers [37]. It was found that with 20 or more highly polymorphic loci, all methods (2 likelihood based and an exclusion-Bayes’ theorem approach) could be used with confidence. Although in this present study the true parentage was not known for the flocks and hence the accuracy could not be determined, a simulation was performed and analysed with Cervus to assess this. This resulted in only 0.13% of progeny incorrectly assigned providing confidence in the assignment method and SNP markers. Furthermore, similar results were obtained for the “Gold Standard” analysis and the Parentage SNP assay using the AgR method, however, performance of the 11 MS marker set was poor.

The Parentage SNP assay presented in this study delivers paternity assignment with confidence; however, it may not be adequate for use in product tracing. This would require further investigation as the risk of incorrectly assigning a sire from a genetic data set that contains many putative sires that are highly likely to include relatives (eg full- and half-sibs) still needs to be determined. Hill et al. [23] using a maximum likelihood approach allowing for genotyping error found that the numbers of loci required to be almost certain to identify the true parent is dependent on the number of other candidates and specifically how many are directly related (ie father or full sibs). They determined that between 100 and 150 SNPs is likely to be sufficient for correct identification. This implies that the 84 parentage SNPs in this study may therefore be underpowered for product tracing via sire. The larger set of 384 SNPs identified by Kijas et al. [8] that reconstitute the clustering of individuals achieved when using the full set of ∼1,500 SNP markers may therefore provide the power required for traceability of lamb to geographic origins (via sire assignment). However, optimal selection of SNPs from this larger SNP set may provide adequate traceability as a third of the markers were found to be polymorphic for all breeds that were tested [8]. A reduced set of markers may therefore be more appealing for a commercial/industry perspective. The Bovine HapMap Consortium [38] found that as few as 50 SNPs were required for proof of identity.

Although the Parentage SNP assay in this present study was trialled on flocks from the NZ sheep industry, the panel is also highly likely to be successful internationally. Sheep populations from NZ, Australia and North America clustered together with the European breeds despite their geographical separation, when genotyped with the ∼1,500 SNP panel [8] from which the SNPs in this study were selected. This highlights the genetic relationship and hence the history of these breeds that were developed in Europe preceding importation into Australia and NZ [39], [40] and also to North America [41]. To provide further information to the international sheep community, SNP polymorphisms within 100 bp upstream or downstream of the Parentage SNPs utilized in this study have been identified in the 73 animals that were sequenced to 12x coverage of the sheep genome (ISGC; www.sheephapmap.org). In addition to the advantages of SNP markers for parentage testing compared to MS markers (eg. abundant, robust, amenable to high throughput, low cost, low genotyping error rate, relatively stable inheritance and low mutation rate), SNPs are easy to standardise between laboratories. The selection of an international set of parentage markers that is recommended to be included in all sheep parentage SNP assays globally would greatly aid traceability of meat products internationally. The 84 parentage panel that has been developed in this study has an overlap of 48 and 34 SNPs in common between the 163 “diverse breed” and 109 “ North American” SNPs sets, respectively [22]. There are 83 SNPs in common between the parentage panel in this study and the 383 Australian CSIRO CRC SNP set [21].

Another important aspect of this study was the development of a DNA extraction method from ear-punch tissue samples required to produce high quality DNA for downstream applications such as the Sequenom system utilized in for the Parentage SNP assay, but also for the Illumina iScan-bead chip and next generation sequencing platforms. This was achieved with more than 2,000 samples genotyped with Illumina iScan-bead chip technology resulting in 98.5% of samples producing call rates of at least 98% and more recently with the production of quality whole genome sequencing data using an Illumina HiSeq 2000. The ability to extract high quality DNA in a low cost high throughput manner suitable for multiple genotyping platforms is an essential component for the uptake of genomics technology by the livestock industry, an area that is evolving rapidly. Furthermore, the SNPs that were selected for inclusion in the Parentage SNP assay are also present on the Illumina Ovine 5K SNP chip allowing integration of the genomic tools utilised by the industry; sires that are genotyped with the Ovine 5K SNP chip for genomic selection therefore will not be required to be re-genotyped with Parentage SNP assay for assignment of progeny.

The utilization of an accurate, robust and high throughput cost competitive Parentage SNP assay will allow implementation of progeny testing on large commercial farms where the improved accuracy of sire assignment and genetic evaluations will increase genetic gain in the sheep industry. When multiple-sire mating is used in farm production systems, paternity testing is essential to identify the sires that are producing progeny with the desired and superior traits. It is also important with single sire mating to avoid incorrect progeny assignment, eg incorrect bonding at birth resulting in misallocation of dam (where lambing occurs in the field). Use of correct pedigree is crucial in obtaining accurate estimates of heritabilities and genetic correlations required for developing selection programs in the NZ sheep industry. This will be of even more importance with breeding operations moving to more extensive environments. The performance of progeny in such environments may have different genetic parameters from those currently used by the industry and therefore there is a requirement for estimating these parameters.

## Conclusion

This study has developed a parentage SNP assay for use in the NZ sheep industry for accurate pedigree assignment that will allow a reduction in breeder input over lambing and has advantages over existing microsatellite based parentage tests. In addition, the SNP assay is suitable for paternity testing. The utilization of an accurate, robust and high throughput cost competitive parentage SNP assay will allow implementation of progeny testing into large commercial farms where the improved accuracy of sire assignment and genetic evaluations will increase genetic gain in the sheep industry and allow better management of inbreeding.

## Supporting Information

Figure S1 **Whole genome sequence data utilizing DNA extracted from the ear-punch tissue high throughput method.** The total bases (A) and coverage (B) obtained for 15 individual samples for the raw (solid bars) and trimmed (hatched bar) data obtained from sequencing Illumina Tru-seq paired end libraries (2x 100 bp reads) on a Illumina HiSeq2000 (at Illumina fast track services, San Diego, CA).
(TIF)

Table S1 **The 98 parentage SNP panel included in the Sequenom Parentage 2 iPlex SNP assay.**
(XLSX)

Table S2 **The oligonucleotide sequences for the Sequenom 2x iPlex 98 Parentage SNP assay.**
(XLSX)

Table S3 **The genotypes observed in the ISGC 73 sequenced animals (diverse breed panel) for the 98 parentage SNPs in Sequenom iPlex assay and neighboring SNPs identified in the 100 nucleotide 5′ and 3′ flanking regions.**
(XLSX)
